# Approach to Formalized Ultrasound Credentialing in a Community Hospital Health System with both Academic and Non-Academic Clinical Settings

**DOI:** 10.51894/001c.12748

**Published:** 2020-06-08

**Authors:** Jeremy Long, Stefan Meyering, Timothy Scheel

**Affiliations:** 1 Emergency Medicine Spectrum Lakeland Health; 2 Emergency Medicine John Peter Smith Hospital; 3 Ultrasound Fellow Denver Health

**Keywords:** medical education, pocus, emergency medicine ultrasound, ultrasonography, ultrasound credentialing

## Abstract

**INTRODUCTION:**

In the US, ultrasound in Emergency Medicine (EM) is widely considered the standard of care in clinical practice amongst most Emergency Department providers. At the authors’ institution and affiliates, there were a variety of health care providers utilizing ultrasound for clinical practice, and their skill levels varied, dependent on training and exposure. As an attempt to standardize credentialing practice and determine need for additional training thresholds, the authors endeavored to perform a skills assessment utilizing both written and clinical based practical assessments.

**METHODS:**

A 7 point questionnaire was administered to a convenience sample of providers requesting formal training information, number of ultrasounds performed, and self-assessed competency. A 10 point written assessment with ultrasound knowledge and clinical application questions was also administered. A subsequent clinical assessment on live humans and models was then performed with multiple stations assessing 15 different instrumentation skills and technique, as well as image interpretation and evaluation.

**RESULTS:**

A total of 23 attending EM board-certified physicians, and four advanced practice providers (PA and NP) took the credentialing assessments scoring an average of 7.3 out of 10 (SD 0.83) for the written assessment. Twenty (71%) of the 28 tested passed the clinical evaluation on their initial attempt. Five (17%) passed on a first remediation. Three (10%) required more than one initial revision attempt. All those who did remediate were able to complete the revision with a passing score.

**CONCLUSIONS:**

Overall, the testing was considered a successful process. This program appears to have offered a level of standardization that was appealing to the credentialing body at our institution. We were able to assess to a level of competence considered standard of care by national credentialing bodies.

## INTRODUCTION

At the Spectrum Health Lakeland community-based hospital system (a three location system with a total of 430 beds), the authors have many physicians and advanced practice providers (i.e. nurse practitioner and physician assistants) (APP) working in a variety of clinical settings. These providers vary in training, comfort, competency, and use of ultrasound. Proficiency with ultrasound has quickly become an essential requirement, as well as standard of care in Emergency Medicine (EM).^1–3^ In 2017 at this healthcare system, a need for specific standardized credentialing for ultrasound privileges was identified during a hospital credentialing meeting with multidisciplinary services including Radiology.

To standardize the credentialing and delineation of clinical privileges, the authors developed a hands-on skills assessment and evaluation of clinical application to certify the use of bedside ultrasound by providers within our health system. Some published methods of achieving credentialing of providers performing ultrasonography in an emergent setting have shown a wide variety amongst department and provider credentialing and practice on a national and global level.^4–7^ Examining a trend amongst institutions providing credentialing has almost always included evaluating assessments of providers regarding cardiac ultrasound, abdominal aortic ultrasound, Focused Assessment with Sonography in Trauma (FAST) and pregnancy or pelvic ultrasonography evaluations.^4,8,9^

The American College of Emergency Physicians (ACEP) and the American Institute of Ultrasound in Medicine (AIUM) have also developed policies and procedures related to quality control, patient education, infection control, and safety guidelines to assist emergency departments in the credentialing and clinical use of ultrasound for hospital EM staff.^1,2,8,10^ These guidelines were not meant to be a substitute for any hospital medical staff credentialing that any legislative, judicial, or regulatory body mandates.^1^

Additionally, these guidelines should provide physicians with evidence of training and requisite competence needed to successfully perform and interpret diagnostic ultrasound examinations in their practice area(s).^8^ For example, the ACEP guidelines recommend completion of 150-to-300 correctly performed and interpreted ultrasound studies or 25-to-50 of a particular exam/subset i.e. E-FAST (Extended Focused Assessment with Sonography of Trauma) gallbladder, OB/GYN, vascular etc.) for a provider to be credentialed.^1^ Additional specialties aside from EM appear to follow guidelines released by the AIUM and an international group for sonography training and credentialing of physicians.^5,8,11^

For this study, we accepted the national ACEP guidelines as a reasonable standard for our intents and purposes as they pertain to EM for credentialing matters. Before this study, the authors had speculated that both a written and hands-on skills assessment would allow a sample of EM providers to demonstrate their proficiency and allow us to identify and correct any problems, including a potential need for additional training to attain adequate credentialing. We also endeavored to utilize the four main ultrasound assessments as mentioned above to generate global competency assessments for our own credentialing study.^4^

## METHODS

This study was conducted to assess providers on their ability to perform cardiac, E-FAST, aorta, and early pregnancy studies by using both a clinical competency evaluation (i.e., ability to acquire the images) as well as a clinical knowledge assessment of images acquired, artifacts, complications, normal and abnormal findings. After IRB approval, all eligible providers were advised regarding how the general topic of study would be assessed one week prior to their assessment and evaluation. A total of nine sessions were completed in order to enroll the total sample.

Observing an opt in recruitment method, participating providers were told that it was considered acceptable to prepare on their own volition without being provided study materials. We did not poll the participants on the level of preparation prior to the assessment. To discern their level of prior training, participants were sent seven-item surveys to investigate their prior ultrasound credentialing experiences. Providers were subsequently asked to complete a 10-item written quiz immediately prior to the final clinical competency evaluation.

### Pre-assessment Survey Questionnaire and Quiz

Both the pre-assessment survey questionnaire and quiz were developed by the authors. The pre-assessment survey included items such as: “Please specify whether you received ultrasound exposure during either graduate (e.g., medical school/NP school/PA school), Post graduate (e.g., Residency/Fellowship), clinical training (i.e., for advanced practice providers or physicians who did not complete formal ultrasound training), Sim lab/Model instruction, or National Training Course/CME workshop exposure.” (See Appendix for full assessment survey)

### Clinical Competency Exam

Each provider was then asked to also perform a series of 15 clinical ultrasounds on live or simulation models involving a complete cardiac exam, E-FAST, early pregnancy exam and aortic ultrasound exam. Their competency levels were assessed on each of the following exam knowledge and techniques:

1. Knowledge of and ability to perform each of the following assessments: Cardiac, E-FAST, Aorta, and Early Pregnancy ultrasound studies.
2. Instrumentation and technique specific to the individual study:
  - Probe placement.
  - Probe orientation.
  - Artifacts or image quality.
  - Image acquisition.
3. Image interpretation and evaluation during each study:
  - Directed questions on what abnormalities they have noted or are being assessed for depending on the specific image or study being acquired.
  - Participants were shown pictures of abnormal findings of multiple studies and asked to interpret the images.

### Scoring

Participants needed to score at least seven out of 10 points on their initial assessment quiz to move to the next clinical competency exam testing phase or else take a similar additional evaluation quiz. For each study in the clinical competency exam, the subject was assigned a score of ‘1’ for each view obtained correctly in each subcategory for a total of four points possible per study (the scoring system was applied to each study with four total studies).

The image interpretation evaluation of abnormalities was the score out of four possible points for each abnormality recognized and correctly defined. Subsequent additional image interpretations also resulted in a total of two possible points. The maximum score possible for the clinical competency exam was therefore 40 points (ten points per study at total of four studies).

If providers performed the clinical competency exam correctly and interpreted the images correctly with a score of 80% or greater (i.e., 32/40), they were considered to have “passed” the examination. If the physician or APP did not pass the clinical competency and/or quiz, they were immediately remediated and shown what they had done incorrectly during their initial clinical exam. The pre-test questionnaire was also reviewed and discussed with the participant and the authors provided brief education regarding any missed answers.

Providers were then directed to another table where they were tested again by another examiner in a similar clinical competency exam format utilizing different models. If they were not able to pass after two remediation attempts, they were not credentialed or asked to test again.

## RESULTS

Analytics were completed by the ultrasound leadership team authors, consisting of the Ultrasound Director, Ultrasound Fellow, and Ultrasound Research Track resident. The total number of participants was 27, consisting of 23 (85%) attending Emergency Medicine board-certified physicians, and four (15%) APP.

### Pre-assessment Survey Questionnaire

Of the 28 participants, the majority 19 (67%) reported having received some level of prior ultrasound training primarily in residency. The minimum time spent of dedicated training for participants was two weeks with a maximum of two months. Other responses consisted of longitudinal training as opposed to dedicated block time (i.e., formal ultrasound rotations or isolated training).

Of the nine (32%) other respondents, one had completed fellowship ultrasound training, four had attended formal ultrasound courses including national courses, university-based training courses or continuing medical education courses. The additional four respondents had received no prior formal training. All respondents who had formal ultrasound training in residency noted the requirements to be at minimum 100 scans, with the maximum of 250 scans as a graduation requirement.

Twenty (71%) respondents had initially indicated that they felt competent to obtain and interpret an ultrasound in their current clinical practice. Most respondents 18 (64%) noted their subjective skill level to be “average,” with eight (29%) estimating their skills to be “below average” or “fair,” and two (7%) noting their skills to be “above average” or “excellent.” All respondents noted they felt competent to perform an ultrasound in a setting where a Radiologist interpreted ultrasound was available for comparison.

Only 16 (57%) respondents indicated that they felt “absolutely comfortable” performing POC (Point of care) ultrasound without the ability to order a comparison study (i.e., formal study obtained by a credentialed ultrasound technologist and interpreted by a Radiologist).

### Quiz

This score was calculated on a ten-point scale. The highest score was 10 (out of 10 correct answers) and the lowest was 3, with an average score of 7.3 (SD 0.83) for all initial quiz attempts. A total of 21 (71%) of the 28 providers passed their first pre-assessment quiz, with the additional six (21%) achieving a passing score after a second attempt. (Figure 1)

### Clinical Competency Exam

Twenty (71%) of the 28 tested providers passed the evaluation on their initial attempt. Five (18%) providers passed after a first remediation. Three (11%) providers required more than one initial revision attempt. All those who did remediate were able to complete the revision with a passing score. (Figure 1) Due to our limited sample size, we did not examine tabulated results delineating performance per sample subgroups (i.e., APP vs Physician).

**Figure:**
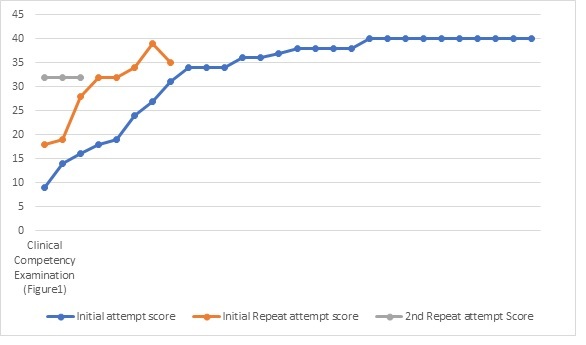
(Figure 1) Scoring of initial competency exam attempts and repeat scores for those who scored < 32 after initial remediation.

## DISCUSSION

Physician ultrasound credentialing is the process of evaluating a provider’s qualifications when considering appointment to the medical staff. Delineation of clinical privileges denotes those specific services and procedures that a physician or APP is deemed qualified to provide or perform.^3,10^ The specific processes for EM provider credentialing must be defined by medical staff and department bylaws, policy, rules, or regulations according to most recent ACEP criteria.^1^ Each member of the medical staff should also be subject to periodic review as part of the performance improvement activities of the organization.^2^

We had concluded that the ACEP had provided good guidelines for the basics of this credentialing process but wanted a way for providers to demonstrate their proficiency with image acquisition and interpretation. We are now performing ongoing quality assessments of each provider with monthly image acquisition evaluations, image quality control, reviews of physician image interpretation and clinical application.

We acknowledge that it can be difficult to accurately gauge whether providers truly know and understand during such credentialing programs, especially across a variety of settings and the presence of residency training programs. It may be important when considering these results that our sample providers had trained at a variety of programs in the country and received various levels of training when examined.^1,10,12^

Without a structured hands-on assessment, it has been found to be difficult to ascertain each provider's individual level of competence with ultrasound.^4,11,12^ It is also difficult to assess during QA processes how frequently attending physicians obtain and accurately interpret images themselves during rushed practice encounters or primarily/solely rely on resident physician’s images interpretations.

To standardize credentialing and delineation of clinical privileges in our three emergency departments, we conducted this pre and post-test survey study comprised of hands-on skills assessments and evaluation procedures for credentialing of bedside ultrasound by providers in our three-hospital health system. We patterned this model after ACEP guidelines after reviewing their competency recommendations and from additional recommendations from other national groups.^1,2,8,11^

The results of our testing project were encouraging. We encountered several providers who tested well with either perfect or near perfect scores. Not unexpectedly these happened to be physicians who were formally trained in ultrasonography through residency teaching and/or ultrasound fellowship experience.

Additionally, the majority of those who were more experienced with ultrasound or fellowship trained in ultrasound tended to complete the exams faster and easier. Again, we did not evaluate the sample to delineate competence between APP and physician groups and acknowledge that this could be evaluated in future larger-sample studies if indicated.

After remediation attempts, most sample physicians and APP’s who completed the assessment met the criteria to be credentialed. For those who scored lower or required remediation attempts, we recommended additional training with in-house formalized ultrasound courses or recommended national training courses of their choosing.^3,9^

One difficulty of completing examinations included the coordination of participant’s many varied shift work and off-time schedules. Since we wanted to make it as painless as possible for the examinees and not take up much of their time outside of work, we came in on several occasions over almost three weeks before or after participants’ shifts to attempt completion of standardized credentialing assessment.

In the future, we plan to send an anonymous survey to participating providers assessing their perceptions of the credentialing process as well as constructive feedback for improved and streamlined process going forward. We have noted prior studies that specified the usage of a follow up questionnaire or poll in our literature search for this study.^13,14^ The following were suggested questions:

1. Did you feel the assessment fairly evaluated your skills with ultrasound as it pertains to use in an emergency department? If not, please explain.
2. Did you feel that your knowledge and competency was increased or assisted by the evaluation?
3. Did you feel that you require more overall ultrasound training in emergency medicine?
4. If you answered yes to the above question would you prefer that to be in the form of in-institution CME/conferences, in-institution training/procedural/SIM labs, out of institution CME/conferences, out of institution training/procedural/SIM labs, or a formal training course in beginning/advanced ultrasound? Please specify.
5. Did you feel that the assessment was standardized enough to be applicable to the various ED settings in which you practice currently for credentialing?
6. Any additional suggests or feedback?

## CONCLUSIONS

As we had residents, fellows, medical and nursing students, APP students, and ultrasound tech/radiology tech students all completing point-of-care ultrasounds at our health system, our administration had concluded that we required a level of standardized ultrasound credentialing for our physicians and APPs for completing and supervising point-of-care ultrasounds in our emergency department settings. Overall, we considered this ultrasound credentialing project to be successful using this assessment and remediation method. The standardized program appeared to participants and our institution to reflect the level of credentialing competence required to deliver care standards by national credentialing bodies.

Based on these results, this method could be sufficient for ultrasound credentialing in additional academic and community settings. We now plan to repeat the credentialing program for any new or returning physician or APP on a bi-annual basis. We will also continue to provide in-house ultrasound training and dedicated ultrasound courses to all those providers who participated in this credentialing program or those who wish to have additional education.

### Quiz

1. What are the views you should obtain in an E-FAST examination?
2. What amount in mL of free intraperitoneal fluid is likely to show a ‘positive result’ on a E-FAST examination?13
3. An aorta greater than what diameter is considered abnormal?
4. When measuring the aorta, do you measure the diameter from internal wall to internal wall or external wall to external wall?
5. What is the first reliable sign of an intrauterine pregnancy on Ultrasound imaging?
6. If you find free fluid in the pelvis of a hypotensive female you are evaluating for possible ruptured ectopic pregnancy, what additional Ultrasound exam/ultrasound views may be helpful to obtain in this scenario?
7. What are the four views of a cardiac study and please note the probe position/direction for each?
8. Which is the best view to obtain to measure/evaluate a pericardial effusion?
9. Name a method to sonographically calculate cardiac contractility or the Left Ventricular Ejection Fraction (LVEF) percentage.
10. Name any two types of Ultrasound specific artifacts which may be noted during an ultrasound exam with explanation of each?

### Clinical Competency Exam

Perform all of the following assessments: Cardiac, E-FAST, Aorta, and Early Pregnancy ultrasound studies.

1. Assessment of the Instrumentation and technique specific to each individual study:
  - Probe placement.
  - Probe orientation.
  - Artifacts or image quality.
  - Image acquisition.
2. Image interpretation and evaluation during each study:
  - Directed questions on what abnormalities they have noted or are being assessed for depending on the specific image or study being acquired.
  - Participant is shown pictures of abnormal findings of multiple studies and asked to interpret the images.

### Conflict of Interest

The authors declare no conflict of interest.
